# Brooke-Spiegler Syndrome With Cervical Spine Lesion

**DOI:** 10.7759/cureus.10982

**Published:** 2020-10-16

**Authors:** Valentina Vasenina, Thomas Cibull, Noam Stadlan

**Affiliations:** 1 Neurosurgery, University of Chicago, Chicago, USA; 2 Pathology and Laboratory Services, NorthShore University HealthSystem, Evanston, USA; 3 Neurosurgery, University of Chicago Pritzker School of Medicine, Chicago, USA; 4 Neurosurgery, NorthShore University HeathSystem, Chicago, USA

**Keywords:** carcinoma, hereditary skin neoplasms, sweat gland neoplasms, adenoid cystic, epidural space

## Abstract

Brooke-Spiegler syndrome (BSS) is a rare hereditary autosomal dominant disorder with variable phenotypic expressivity that results in a variety of benign cutaneous face, scalp, and neck tumors with a histology profile of cylindroma, spiradenoma and trichoepithelioma. Reports of lymph node and distant metastasis are scarce. We present the first case of Brooke-Spiegler syndrome with metastasis to the cervical spine. An 86-year-old female with Brooke-Spiegler syndrome presented to the clinic with a finding of cervical spine lesion involving vertebral body, prevertebral, paraspinal, foraminal, and epidural spaces. The histopathology of the lesion showed benign cylindroma. Considering the location of the lesion and local invasion of neural structures, the malignant transformation of existing tumors could not be excluded. Brooke-Spiegler syndrome rarely presents with malignant transformation and distant metastatic spread. It is important to be aware of these rare cases while monitoring the disease and addressing clinical symptoms. This is to our knowledge the first case of metastatic spread of the cylindroma to the cervical spine resulting in local bone destruction and neural elements compromise. Physicians should be aware of this rare possibility.

## Introduction

Brooke-Spiegler syndrome (BSS) is a rare autosomal dominant disorder with a variable phenotypic expression, which can present with multiple cutaneous adnexal neoplasms of the scalp, face, and neck with less frequent trunk and extremities involvement. Lesions begin to appear in late childhood-late adolescence and slowly increase in size and number. It is more prevalent in females [1-3]. Histologically, the defining lesions of Brooke-Spiegler syndrome are cylindromas, spiradenomas, and trichoepitheliomas. Multiple familial trichoepithelioma and familial cylindromatosis are phenotypic variants of Brooke-Spiegler syndrome. In addition to above-mentioned skin lesions, patients with Brooke-Spiegler syndrome may develop salivary gland tumors, basal cell carcinomas, trichoblastomas, milia, organoid nevi. Cylindromatosis gene (CYLD) tumor-suppressor gene mutation on chromosome 16 is detected in about 80-85% of patients with the classical Brooke-Spiegler presentation [4]. The terminology to refer to this condition and its phenotypic variants is changing and is now more commonly referred to as CYLD cutaneous syndrome (CCS) [5]. 

Malignant transformation of cutaneous neoplasms occurs in 5-10 % of the patients [6]. Reports of lymph node and distant metastatic spread are extremely rare, though there are reports of pulmonary cylindroma [5]. We present the first case of Brooke-Spiegler syndrome with metastasis to the cervical spine. 

## Case presentation

An 86-year-old female initially presented to our clinic with complaint of intermittent left arm pain and left fingers paresthesia in dermatomal distribution and left lower extremity pain for one year. The patient’s medical history was significant for Brooke-Spiegler syndrome with multiple scalp lesions resected throughout her life and parotid basal cell adenoma. No changes to the scalp lesions were noted. The patient had no neurological deficits on physical exam.

**Figure 1:**
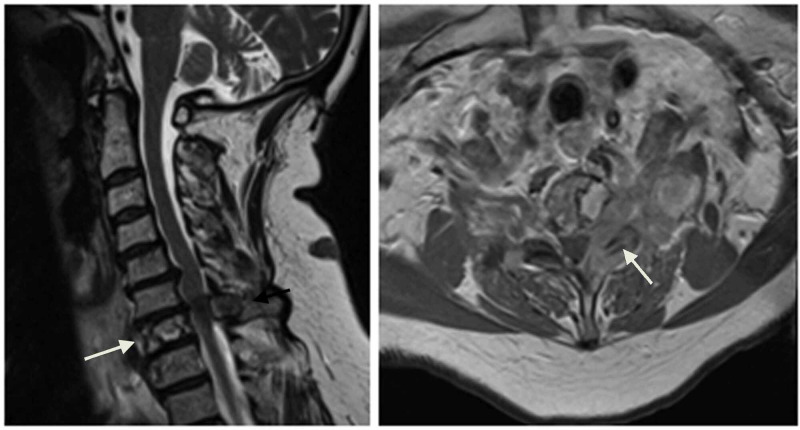
Left: Sagittal T2-weighted image of the cervical spine showing soft tissue mass invading C7 vertebral body and spinous process (arrow). Right: Axial post-contrast T1 weighted image of the cervical spine showing tumor extension into C67 neural foramen (arrow).

CT of the cervical spine demonstrated lytic lesion of C7 vertebral body. MRI of the cervical spine showed 4.7 x 4.9 cm destructive heterogeneous soft tissue mass involving C7 vertebral body, pedicle and spinous process with extension into left C67, C7T1 neuroforamina, left C7 epidural space and left paravertebral/prevertebral space (Figure 1). The left C7 and C8 nerve root compression was consistent with patient’s left arm radicular symptoms. The etiology of the left leg pain was unknown. 

**Figure 2:**
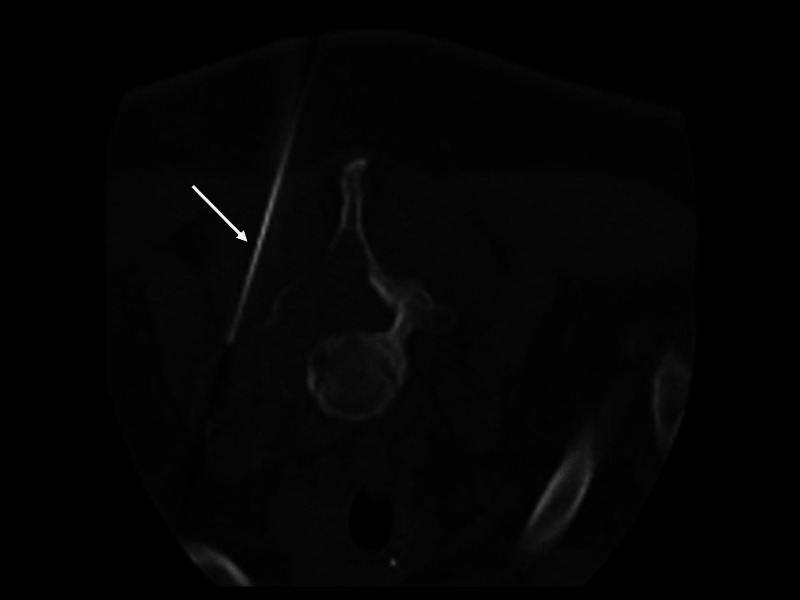
Needle biopsy tract (arrow).

Needle biopsy of the lesion (Figure 2) was consistent with skin adnexal neoplasm in the spectrum of cylindroma/spiradenoma.

**Figure 3:**
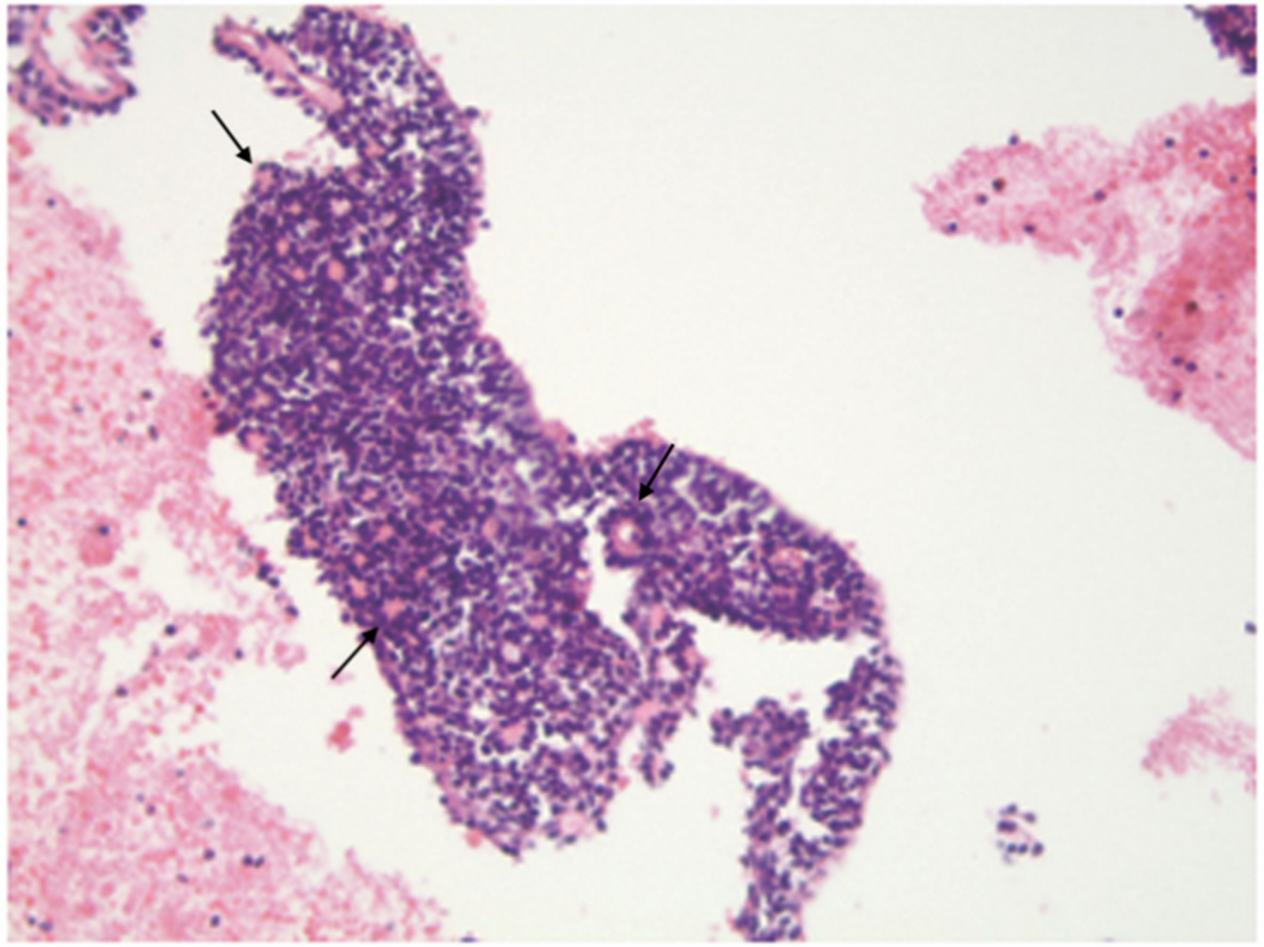
Cylindroma. Tumor comprised of small basaloid cells admixed with some larger cells. There are scattered small, duct-like structures (arrow).

Though no malignant features were detected on histologic examination (Figure 3), the unusual location of the lesion and local invasion of neural structures raised concern for malignant transformation of existing cylindromas. No genetic analysis was done. The patient was subsequently lost to follow up.

## Discussion

Malignant cylindroma (cylindrocarcinoma) is an extremely rare entity with distant metastatic spread being an exceptional event. The age of the patients with cylindrocarcinoma ranges from 50 to 96 years old; females affected more often [7]. Some of the authors report on greater preponderance for malignant transformation in the context of Brooke-Spiegler syndrome and its phenotypic variant, familial cylindromatosis [8]. The malignant tumors are usually distinguished from the benign lesions by rapid growth, ulceration, bleeding, and pain [9]. Seventy-two cases of malignant cylindromas have been described in the literature. There are less than 20 cases of lymph node and distant metastatic spread of malignant cylindromas [10]. The malignant tumor most likely spreads along draining lymphatic vessels to the associated regions [11]. The regions of distant metastasis include cervical and visceral lymph nodes, lung, and pleura [10, 12]. Direct invasion of the cranial bone and intracranial compartment has also been observed with malignant cylindromas of the scalp [11,13]. Benign cylindroma eroding through the skull vault has only been reported once [14].

In summary, this case demonstrates a unique behavior of histologically benign cylindroma with new site of metastatic occurrence and local invasion. This is the first case reported in the literature of this pattern of behavior.

## Conclusions

To our knowledge, this is the first case of metastatic spread of the benign cylindroma to the cervical spine resulting in local bone destruction and compromised neural elements. Physicians taking care of patient populations with Brooke-Spiegler syndrome should be aware of this rare possibility.
